# Postoperative risks of type 2 diabetes in elderly hip fracture patients: a propensity score-matched study

**DOI:** 10.1007/s00774-025-01624-9

**Published:** 2025-07-01

**Authors:** Yu Mori, Kunio Tarasawa, Hidetatsu Tanaka, Naoko Mori, Kiyohide Fushimi, Toshimi Aizawa, Kenji Fujimori

**Affiliations:** 1 Department of Orthopaedic Surgery, Tohoku University Graduate School of Medicine, 1-1 Seiryo-machi, Aoba-Ku, Sendai, Miyagi 980-8574 Japan; 2 Department of Health Administration and Policy, Tohoku University Graduate School of Medicine, 2-1 Seiryo-machi, Aoba-Ku, Sendai, Miyagi 980-8574 Japan; 3 Department of Radiology, Akita University Graduate School of Medicine, 1-1-1 Hondo, Akita, Akita 010-8543 Japan; 4 Department of Health Policy and Informatics, Institute of Science Tokyo, 1-5-45 Yushima, Bunkyo-Ku, Tokyo, 113-8519 Japan

**Keywords:** Postoperative complications, Type 2 diabetes, Hip fracture, Mortality, Nationwide database

## Abstract

**Introduction:**

Type 2 diabetes is associated with an increased risk of fragility fractures, even in individuals with normal or high bone mineral density. However, the impact of type 2 diabetes on postoperative outcomes after hip fracture surgery in elderly Japanese patients remains unclear. This study evaluated the association between type 2 diabetes and postoperative complications, including in-hospital mortality, using a nationwide database in Japan.

**Materials and methods:**

A retrospective cohort study was conducted using the Diagnosis Procedure Combination (DPC) database from April 2016 to March 2022. Patients aged ≥ 65 years who underwent hip fracture surgery were included. Propensity score matching (1:1) was performed to adjust for confounders. Logistic regression analyses were used to assess associations between type 2 diabetes and outcomes.

**Results:**

Of the 474,293 eligible patients included in this study, 18.5% were identified as having comorbid type 2 diabetes. Following 1:1 propensity score matching, the final analytic cohorts each comprised 83,283 patients. Although statistically significant, the presence of type 2 diabetes was associated with only modest increases in the risks of postoperative myocardial infarction (risk difference [RD]: 0.0007), cognitive dysfunction (RD: 0.0029), and in-hospital mortality (RD: 0.0045), with all comparisons yielding p-values of less than 0.0001. Additionally, the length of hospital stay was longer among patients with type 2 diabetes.

**Conclusions:**

Although the absolute risk differences were small, type 2 diabetes remains an independent risk factor for adverse postoperative outcomes following hip fracture surgery in elderly Japanese patients. Tailored perioperative strategies may help optimize outcomes in this vulnerable population.

**Supplementary Information:**

The online version contains supplementary material available at 10.1007/s00774-025-01624-9.

## Introduction

Hip fractures constitute a frequent orthopedic injury in the elderly and are associated with considerable morbidity and mortality [1, 2]. The global incidence of hip fractures is projected to increase to approximately 4.5 million cases by the year 2050 [3]. In the United States, the expanding elderly population has contributed to an increase in proximal femur fractures, with the number of cases anticipated to double from 250,000 in 1990 to 500,000 by 2040 [4]. Similarly, in Japan, the aging society has resulted in an estimated 13 million individuals being affected by osteoporosis [5], and the annual number of proximal femur fractures is estimated to be approximately 250,000 [6]. Consequently, osteoporosis-related proximal femur fractures have emerged as a major global health concern, transcending national and regional boundaries. Notably, more than 90% of these fractures occur in individuals aged 65 years or older, among whom comorbidities tend to accumulate with age. The 30-day mortality rate following a hip fracture is reported to range from 4.0% to 5.4% [7], while the 1-year mortality rate approaches 25% [8].

Type 2 diabetes is a representative lifestyle-related disease, and its prevalence has been rapidly increasing in Japan due to the Westernization of diet and lifestyle. In recent years, epidemiological and clinical studies have identified type 2 diabetes as a risk factor for osteoporotic fractures [9, 10]. According to the previous report, 13.6% of men and 8.5% of women aged 60 years and older in Japan have type 2 diabetes, with prevalence increasing with age [11]. In type 2 diabetes patients, the osteogenic differentiation of mesenchymal stem cells is suppressed, and the inflammatory response essential for bone healing is also impaired. Oxidative stress and chronic inflammation contribute to these dysfunctions, leading to delayed bone regeneration [12, 13]. Osteogenic differentiation of mesenchymal stem cells plays a critical role in fracture healing, and an appropriately regulated inflammatory environment is essential for tissue repair [14, 15]. Therefore, in type 2 diabetes patients, there is concern regarding an increased risk of fragility fractures due to impaired bone regeneration.

Type 2 diabetes has been associated with an elevated risk of mortality following hip fracture. The comorbidity of type 2 diabetes has been identified as a contributing factor to the increased risk of postoperative complications following hip fracture. Previous studies have reported a 1-year mortality rate of 23.3% in patients with type 2 diabetes compared to 17.1% in those without, with an odds ratio (OR) of 1.36 (95% Confidence Interval [CI]: 1.03–1.80) [16]. Furthermore, type 2 diabetes-related mortality has been quantified with an adjusted risk ratio of 1.17 (95% CI: 1.09–1.25) [17].

Studies using the Diagnosis Procedure Combination (DPC) database of Japanese hip fracture patients have reported the postoperative complication risks associated with hip fractures in the elderly and total hip arthroplasty [18–25]. Despite growing evidence linking type 2 diabetes to adverse health outcomes, the impact of type 2 diabetes on postoperative outcomes after hip fracture surgery in elderly Japanese patients remains unclear, necessitating population-specific research. This study aims to investigate postoperative complications and in-hospital mortality in patients aged 65 years and older, with and without type 2 diabetes, using a large Japanese hip fracture database and adjusting for potential confounders.

## Materials and methods

### Study design

This retrospective cohort study was conducted in accordance with the ethical principles of the Declaration of Helsinki and was approved by the Institutional Review Board of Tohoku University (approval No. 2024–1-1026).

### Data source

The data were retrospectively collected from Japan's nationwide administrative DPC reimbursement system database [26]. Comprehensive informed consent was obtained from all patients upon admission, covering both their agreement to the proposed treatment methods and permission for the academic use of data collected during their care. No personally identifiable information is included in this study. All variables used in the analysis were derived from this data source. Cases with missing values for any relevant clinical variables were excluded to ensure data completeness and accuracy. No imputation methods were applied, and only cases with complete data were included in the final analysis.

### Inclusion and exclusion criteria

The sample size for this study was determined based on a fixed study period rather than a priori power calculation. Patients were identified from a nationwide survey of hospitals participating in the Japanese DPC system, covering the period from April 2016 to March 2022. Approximately 1,100 DPC-participating hospitals consistently submitted medical records and were approved for inclusion in this study. The analysis included patients who underwent surgical treatment for hip fractures at these hospitals across Japan. This clinical investigation focused on elderly patients aged 65 years and older, specifically examining the incidence of postoperative complications and short-term in-hospital mortality among those with type 2 diabetes. Patients under 65 years of age and those treated conservatively for hip fractures were excluded. All eligible cases meeting the inclusion criteria during the study period were included to ensure a comprehensive representation of the target population.

### Exposure of interest

This study examined patients who underwent surgical treatment for hip fractures, with a focus on comparing the incidence of postoperative complications during hospitalization between those with and without type 2 diabetes. Patients with type 2 diabetes were identified based on diagnostic codes (ICD-10 codes E11, E14, R730). Information on the duration and treatment of type 2 diabetes was not included in the analysis.

### Outcomes of interest

The primary outcomes of this study were the incidence of postoperative complications and in-hospital mortality. These outcomes were selected due to their clinical relevance in evaluating the short-term risks associated with hip fracture surgery in elderly patients. The secondary outcomes were length of hospital stay and transfusion volume, which reflect healthcare utilization and perioperative management burden. The postoperative complications assessed in this study included venous thromboembolism, cardiac infarction, urinary tract infection, cognitive dysfunction, pneumonia, and in-hospital mortality. Postoperative cognitive dysfunction was identified using ICD-10 codes F010, F011, F012, F019, F03, F107, G238, G300, G301, G308, G309, G310, and G318, which encompass various forms of cognitive impairment and delirium occurring during the postoperative period, as previously described [19]. All outcomes were identified based on diagnostic codes. As mortality was included as one of the outcomes, the assessment period for all complications covered the entire duration of hospitalization. Secondary outcomes included length of hospital stay, total volume of perioperative blood transfusion, and the use of anti-osteoporotic medications.

### Covariates

Covariates included age, sex, body mass index (BMI), type of anesthesia, fracture classification, surgical procedure, timing of surgery (categorized as within 2 days of admission or on/after day 3), comorbidities, including hypertension, ischemic heart disease, cerebrovascular disease, chronic renal disease, chronic lung disease, cognitive impairment. In addition, the Charlson Comorbidity Index, calculated from ICD-10 codes based on the original algorithm by Charlson et al. (1987) [27], was used to provide an overall assessment of comorbidity burden but was not included in the propensity score model to avoid redundancy with individual comorbid conditions. Age was included as a separate covariate in the analysis. Hip fractures were classified according to ICD-10 codes as follows: S7200 for femoral neck fractures, S7210 for trochanteric fractures, and S7220 for subtrochanteric fractures. Patients were selected for inclusion in the hip fracture cohort based on registry data, with eligibility determined by meeting all three of the following criteria: (1) principal diagnosis, (2) primary reason for hospital admission, and (3) condition associated with the highest medical resource utilization.

### Propensity score matching

To reduce confounding in the comparison of postoperative complications between patients with and without type 2 diabetes, a 1:1 propensity score matching was conducted based on clinically relevant covariates. The matching model included age, sex, BMI, timing of surgery, type of anesthesia, hip fracture classification, surgical procedure, and various comorbidities, including hypertension, ischemic heart disease, cerebrovascular disease, chronic renal disease, chronic lung disease, cognitive impairment, and the Charlson Comorbidity Index. Propensity scores were estimated using logistic regression, and nearest-neighbor matching without replacement was applied using a caliper width of 0.2 times the standard deviation of the propensity score estimates. Covariate balance after matching was evaluated using standardized mean differences (SMDs), with an SMD of less than 0.1 considered indicative of adequate balance. Multivariate logistic regression analyses were performed within the matched cohort to identify independent associations with outcomes, incorporating the covariates used in the propensity score model. The model's discriminatory performance was assessed using the C-statistic. This approach ensured a balanced comparison between type 2 diabetes and non-diabetes patients by minimizing baseline differences through adjustment for key confounders.

**Fig. 1:**
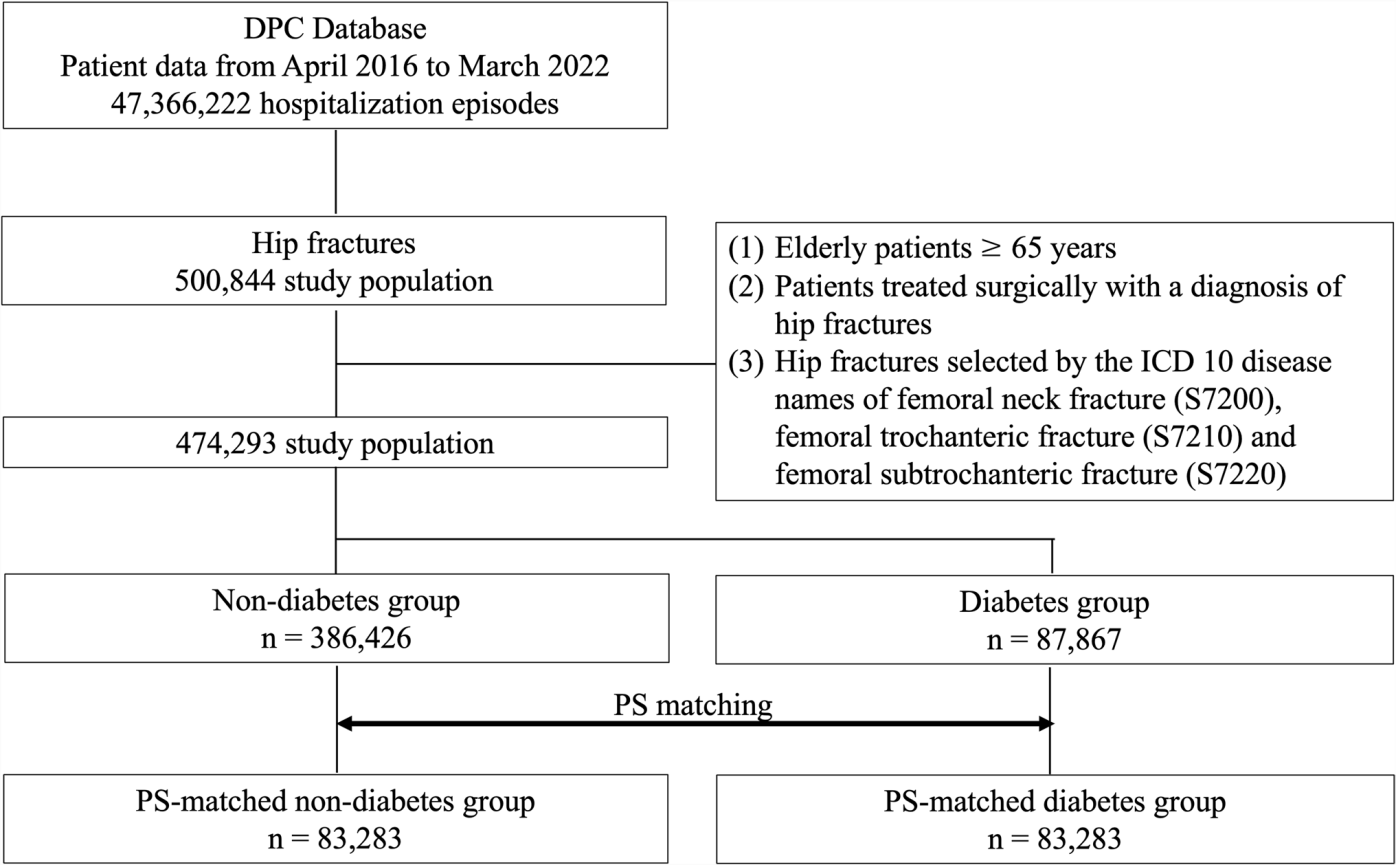
Flow diagram of patient selection for type 2 diabetes and non-diabetes patients with hip fracture and propensity score (PS) matching. This diagram shows the method for extracting target patients from the DPC database and PS matching for type 2 diabetes and non-diabetes patients with hip fractures

Figure 1 illustrates a schematic overview of the patient selection process. From the dataset covering the period between April 2016 and March 2022, a total of 474,293 patients met the predefined inclusion and exclusion criteria. Of these, 386,426 were classified as non-diabetes patients and 87,867 as type 2 diabetes patients. It was demonstrated that 18.5% of hip fracture cases among elderly individuals aged 65 years and older were associated with type 2 diabetes. Following 1:1 propensity score matching based on age, sex, BMI, various comorbidities, Charlson Comorbidity Index, timing of surgery, use of general anesthesia, type of hip fracture, and surgical procedure, matched cohorts were established, each comprising 83,283 patients in the type 2 diabetes and non-diabetes groups.

Table 1 presents the baseline characteristics of patients with and without type 2 diabetes who underwent surgical treatment for hip fractures. Prior to propensity score matching, SMDs for sex, age, BMI, hypertension, ischemic heart disease, and timing of surgery exceeded 0.1, indicating notable imbalances between the groups. The type 2 diabetes group had a higher proportion of male patients, was generally younger, and exhibited higher BMI values. Furthermore, the type 2 diabetes group exhibited a higher prevalence of hypertension and ischemic heart disease, as well as a lower proportion of patients undergoing early surgery within 2 days of admission. Additionally, general anesthesia was more commonly used, and femoral neck fractures were the most prevalent fracture type among type 2 diabetes patients. Following 1:1 propensity score matching, all covariates—age, sex, BMI, comorbidities, Charlson Comorbidity Index, timing of surgery, use of general anesthesia, fracture type, and surgical procedure—achieved SMDs below 0.1, confirming adequate balance between the groups. The C-statistic for the propensity score model was 0.781, indicating good discriminatory ability.

**Table 1:** Characteristics of patients before and after propensity score matching

	Before PS matching			After PS matching		
	Non-diabetes	Diabetes	SMD	Non-diabetes	Diabetes	SMD
n	386,426	87,867		83,283	83,283	
Sex						
Men	79,358 (20.5%)	23,806 (27.1%)	0.15	23,034 (27.7%)	226,669 (27.2%)	0.009
Women	307,068 (79.5%)	64,061 (72.9%)		60,249 (72.3%)	60,614 (72.8%)	
Age	84.8 ± 7.7	82.6 ± 7.5	0.29	82.5 ± 8.0	82.6 ± 7.5	0.014
Body mass index	20.3 ± 5.2	21.5 ± 4.5	0.25	21.4 ± 4.7	21.5 ± 4.5	0.024
Charlson comorbidity index	1.2 ± 1.3	1.2 ± 1.3	0.001	1.2 ± 1.3	1.2 ± 1.3	0.009
Hypertension	144,021 (37.3%)	38,791 (44.1%)	0.14	37,188 (44.7%)	36,898 (44.3%)	0.007
Ischemic heart disease	28,402 (7.4%)	9764 (11.1%)	0.13	9533 (11.4%)	9358 (11.2%)	0.007
Cerebrovascular disease	37,859 (9.8%)	10,873 (12.4%)	0.082	10,423 (12.5%)	10,347 (12.4%)	0.003
Chronic renal disease	20,203 (5.2%)	5360 (6.1%)	0.039	5217 (6.3%)	5127 (6.2%)	0.004
Chronic lung disease	5974 (1.6%)	1196 (1.4%)	0.015	1095 (1.3%)	1141 (1.4%)	0.005
Cognitive impairment	87,357 (22.6%)	16,864 (19.2%)	0.086	15,636 (18.8%)	15,874 (19.1%)	0.007
Surgery within 2 days	188,690 (48.8%)	38,246 (43.5%)	0.11	35,961 (43.2%)	36,211 (43.4%)	0.006
General anesthesia	232,390 (60.1%)	56,025 (63.8%)	0.075	53,479 (64.2%)	53,353 (64.1%)	0.003
Fracture classification						
Femoral neck	193,408 (50.1%)	45,016 (51.2%)	0.019	43,551 (52.3%)	42,757 (51.3%)	0.015
Trochanteric	184,804 (47.8%)	40,837 (46.5%)		37,735 (45.3%)	38,624 (46.4%)	
Subtrochanteric	8214 (2.1%)	2014 (2.3%)		1997 (2.4%)	1902 (2.3%)	
Surgical procedure						
ORIF	244,175 (63.2%)	54,275 (61.8%)	0.029	51,476 (61.8%)	51,389 (61.7%)	0.002
Hip arthroplasty	142,251 (36.8%)	33,592 (37.1%)		31,807 (38.2%)	31,894 (38.3%)	
ORIF						
Femoral neck	52,917 (21.7%)	11,847 (21.8%)	0.002	12,137 (23.6%)	11,264 (21.9%)	0.029
Trochanteric	183,139 (75.0%)	40,434 (74.5%)		37,368 (72.6%)	38,243 (74.4%)	
Subtrochanteric	8119 (3.3%)	1994 (3.7%)		1971 (3.8%)	1882 (3.7%)	
Hip arthroplasty						
Bipolar hemiarthroplasty	137,060 (96.4%)	32,369 (96.4%)	0.001	30,245 (95.1%)	30,728 (96.3%)	0.059
Total hip arthroplasty	5191 (3.6%)	1223 (3.6%)		1562 (4.9%)	1166 (3.7%))	

One-to-one PS matching was performed

Data is shown as mean ± standard deviation

*PS* means propensity score, *SMD* means standard mean difference, *ORIF* means open reduction and internal fixation

### Statistical analyses

Data are expressed as mean ± standard deviation. Comparisons between the type 2 diabetes and non-diabetes groups were conducted using the χ^2^ test for categorical variables and Student's t-test for continuous variables. Univariate logistic regression analysis was performed to evaluate severe in-hospital complications and in-hospital mortality. Although SMDs indicated adequate balance between the groups after propensity score matching, a sensitivity analysis was conducted to account for the potential influence of unmeasured confounders. In this analysis, the caliper width for propensity score matching was tightened from 0.2 to 0.05, and similar risks of postoperative complications and in-hospital mortality were observed under these more stringent matching conditions, suggesting the robustness of our findings. To further confirm the consistency of the results, subgroup analyses were performed within the matched cohort by stratifying patients according to fracture type, specifically femoral neck fractures and trochanteric fractures. Survival differences between groups were assessed using the log-rank test. In consideration of the large sample size, a stringent significance level was adopted. All statistical tests were two-tailed, and a p-value of less than 0.001 was considered statistically significant. Statistical analyses were performed using JMP version 17 (SAS Institute Inc., Cary, NC, USA).

## Results

Table 2 presents the findings related to the use of anti-osteoporotic medications. Bisphosphonates and active vitamin D preparations were the most commonly prescribed agents in both the type 2 diabetes and non-diabetes groups. Prior to propensity score matching, the use of bisphosphonates, teriparatide, and denosumab was comparable between the groups. Notably, the use of active vitamin D preparations and selective estrogen receptor modulators (SERMs) was lower in the type 2 diabetes group, a trend that remained consistent following propensity score matching. Active vitamin D preparations were widely utilized across both groups; however, the use of eldecalcitol continued to be lower in the type 2 diabetes group after matching. Although the use of alfacalcidol was initially lower in the type 2 diabetes group, these differences were no longer observed after matching. The use of SERMs continued to be lower in the type 2 diabetes group after matching.

**Table 2:** Comparison of osteoporosis treatment between type 2 diabetes and non-diabetes groups before and after propensity score matching

	Before PS-matching			After PS-matching		
	Non-diabetes	Diabetes	*p*-value	Non-diabetes	Diabetes	*p*-value
Daily bisphosphonates	826 (0.2%)	175 (0.2%)	0.39	197 (0.2%)	165 (0.2%)	0.09
Weekly bisphosphonates	27,961 (7.2%)	6347 (7.2%)	0.89	5978 (7.2%)	6064 (7.3%)	0.42
Monthly bisphosphonates (oral)	10,035 (2.6%)	2167 (2.5%)	0.027	2121 (2.6%)	2003 (2.4%)	0.06
Monthly bisphosphonates (iv)	2828 (0.7%)	585 (0.7%)	0.037	589 (0.7%)	561 (0.7%)	0.41
Yearly bisphosphonates (iv)	627 (0.2%)	130 (0.2%)	0.34	138 (0.2%)	125 (0.2%)	0.42
Daily teriparatide	4164 (1.1%)	922 (1.1%)	0.46	943 (1.1%)	874 (1.1%)	0.11
Weekly teriparatide	2470 (0.6%)	504 (0.6%)	0.026	549 (0.7%)	476 (0.6%)	0.02
Biweekly teriparatide	647 (0.2%)	123 (0.1%)	0.068	163 (0.2%)	115 (0.1%)	0.004
Denosumab	1480 (0.4%)	317 (0.4%)	0.33	295 (0.4%)	296 (0.4%)	0.97
Eldecalcitol	36,173 (9.4%)	7222 (8.2%)	< 0.0001*	7433 (8.9%)	6844 (8.2%)	< 0.0001*
Alfacalcidol	41,131 (10.6%)	8786 (10.0%)	< 0.0001*	8507 (10.2%)	8330 (10.0%)	0.15
SERM	5701 (1.5%)	1061 (1.2%)	< 0.0001*	1180 (1.4%)	1007 (1.2%)	0.0002*

One-to-one PS matching was performed

*PS* means propensity score, *SERM* means selective estrogen receptor modulator

*p-values of < 0.001 are considered significant by the χ^2^ test

Table 3 summarizes the use of anticoagulant and antiplatelet agents administered for venous thromboembolism prevention and management of comorbidities. In both groups, approximately 55% of patients received either anticoagulant or antiplatelet therapy. Edoxaban was the most commonly used agent, followed by aspirin. There were no significant differences in the usage rates of any of these medications between the type 2 diabetes and non-diabetes groups. This trend remained consistent before and after propensity score matching.

**Table 3:** Comparison of antithrombotic therapies before and after propensity score matching

	Before PS matching			After PS matching		
	Non-diabetes	Diabetes	*p*-value	Non-diabetes	Diabetes	*p*-value
Edoxaban	102,109 (26.4%)	23,232 (26.4%)	0.92	22,168 (26.6%)	22,044 (26.5%)	0.49
Fondaparinux	5173 (1.3%)	1165 (1.3%)	0.77	1136 (1.4%)	1108 (1.3%)	0.55
Enoxaparin	14,461 (3.7%)	3314 (3.8%)	0.68	3087 (3.7%)	3123 (3.7%)	0.64
Aspirin	46,724 (12.1%)	10,441 (11.9%)	0.09	9919 (12.0%)	9909 (11.9%)	0.94
Warfarin	14,883 (3.9%)	3391 (3.9%)	0.91	3228 (3.9%)	3200 (3.8%)	0.72
Clopidogrel	21,378 (5.5%)	4855 (5.5%)	0.93	4655 (5.6%)	4631 (5.6%)	0.79
Apixaban	11,491 (3.0%)	2664 (3.0%)	0.23	2480 (3.0%)	2526 (3.0%)	0.51

One-to-one PS matching was performed

*PS* means propensity score

*p-values of < 0.001 are considered significant by the χ^2^ test

Table 4 presents the results of the comparative analysis of complication rates between the type 2 diabetes and non-diabetes groups. Prior to propensity score matching, the incidence rates of myocardial infarction, postoperative cognitive dysfunction, and in-hospital mortality were significantly higher in the type 2 diabetes group, whereas no significant differences were observed between the groups in the rates of venous thromboembolism, urinary tract infection, or pneumonia. After propensity score matching, the type 2 diabetes group continued to show significantly higher rates of myocardial infarction, postoperative cognitive dysfunction, and in-hospital mortality. The corresponding risk differences (RDs) were 0.0007 (95% CI: 0.0004–0.0011) for myocardial infarction, 0.0029 (95% CI: 0.0019–0.0040) for postoperative cognitive dysfunction, and 0.0045 (95% CI: 0.0031–0.0059) for in-hospital mortality (Table 5). Notably, there was no increased risk of venous thromboembolism in the type 2 diabetes group, even after matching. While the in-hospital mortality rate was significantly higher in the type 2 diabetes group (2.0%) compared to the non-diabetes group (1.5%), the absolute risk difference was small (RD: 0.0045), suggesting that the clinical impact may be limited despite statistical significance (Table 4, 5).

**Table 4:** Comparison of complications before and after propensity score matching

	Before PS matching			After PS matching		
	Non-diabetes	Diabetes	*p*-value	Non-diabetes	Diabetes	*p*-value
Venous thromboembolism	17,664 (4.6%)	3993 (4.5%)	0.73	4118 (4.9%)	3810 (4.6%)	0.0004*
Myocardial infarction	309 (0.08%)	121 (0.14%)	< 0.0001*	59 (0.07%)	117 (0.14%)	< 0.0001*
Urinary tract infection	11,775 (3.1%)	2741 (3.1%)	0.26	2399 (2.9%)	2611 (3.1%)	0.002
Cognitive dysfunction	5342 (1.4%)	1380 (1.6%)	< 0.0001*	1049 (1.3%)	1289 (1.6%)	< 0.0001*
Pneumonia	12,311 (3.2%)	2784 (3.2%)	0.79	2492 (3.0%)	2625 (3.2%)	0.059
In-hospital mortality	6802 (1.8%)	1707 (1.9%)	0.0002*	1249 (1.5%)	1620 (2.0%)	< 0.0001*
Length of hospitalization (days)	35.0 ± 27.5	37.0 ± 30.0	< 0.0001*	35.9 ± 27.9	37.2 ± 30.3	< 0.0001*
Blood transfusion Day 0 (unit)	0.46 ± 1.07	0.41 ± 1.04	< 0.0001*	0.42 ± 1.04	0.41 ± 1.04	0.047
Blood transfusion Day 1 (unit)	0.32 ± 0.84	0.28 ± 0.80	< 0.0001*	0.28 ± 0.80	0.28 ± 0.80	0.69

One-to-one PS matching was performed

*PS* means propensity score

*p-values of < 0.001 are considered significant by the χ^2^ test and Student’s t–test

**Table 5:** Risk differences in postoperative complications associated with type 2 diabetes before and after propensity score matching

	Before PS matching		After PS matching	
	Risk difference (95% CI)	*p*-value	Risk difference (95% CI)	*p*-value
Venous thromboembolism	− 0.0003 (− 0.0018 to 0.0013)	0.73	− 0.0037 (− 0.0057 to − 0.0016)	0.0004*
Myocardial infarction	0.0006 (0.0003–0.0008)	< 0.0001*	0.0007 (0.0004–0.0011)	< 0.0001*
Urinary tract infection	0.0007 (− 0.0006 to 0.0020)	0.26	0.0025 (0.0009–0.0042)	0.002
Cognitive dysfunction	0.0018 (0.001–0.0028)	< 0.0001*	0.0029 (0.0018–0.0040)	< 0.0001*
Pneumonia	− 0.0002 (− 0.0015 to 0.0011)	0.79	0.0016 (− 0.00006 to 0.0032)	0.059
In-hospital mortality	0.0018 (0.0008–0.0028)	0.0002*	0.0045 (0.0032–0.0057)	< 0.0001*

*CI* means confidence interval

^*^p-values of < 0.001 are considered significant by the χ^2^ test

Length of hospital stay showed a consistent trend before and after propensity score matching. After matching, the mean hospital stay was 37.2 ± 30.3 days in the type 2 diabetes group and 35.9 ± 27.9 days in the non-diabetes group, indicating a longer hospitalization in the type 2 diabetes group. Regarding perioperative blood transfusion, the volume was higher in the non-diabetes group before matching, but no significant difference was observed between the groups after matching (Table 4). The subgroup analysis for femoral neck fractures yielded results consistent with the main analysis. In contrast, for trochanteric fractures, postoperative myocardial infarction, and cognitive dysfunction showed a trend toward significance but did not meet the predefined threshold for statistical significance. However, in-hospital mortality was significantly higher in the type 2 diabetes group in both femoral neck and trochanteric fracture subgroups. Length of hospital stay showed similar results across both subgroups. However, in the trochanteric fracture subgroup, the type 2 diabetes group had a significantly lower transfusion volume compared to the non-diabetes group (Supplemental Table 1).

Table 6 presents the results of univariate logistic regression analyses conducted to evaluate the impact of type 2 diabetes on postoperative complications and in-hospital mortality following hip fracture surgery. In the univariate analysis, type 2 diabetes was identified as a significant risk factor for myocardial infarction, postoperative cognitive dysfunction, and in-hospital mortality. The OR for type 2 diabetes in relation to in-hospital mortality was 1.303 (95% confidence interval [CI]: 1.209–1.404, p < 0.0001). Similarly, the ORs for type 2 diabetes with respect to postoperative myocardial infarction and cognitive dysfunction were 1.984 (95% CI: 1.451–2.714, p < 0.0001) and 1.232 (95% CI: 1.135–1.337, p < 0.0001), respectively. For the sensitivity analysis, propensity score matching was performed using a caliper width of 0.05, resulting in a matched cohort of 833,265 pairs. Univariate logistic regression analysis demonstrated similar trends in postoperative complications and in-hospital mortality, supporting the robustness of the present study's findings.

**Table 6:** Univariate logistic regression analysis of postoperative complications associated with type 2 diabetes after propensity score matching using two different caliper widths for sensitivity analysis

	Caliper widths of 0.2		Caliper widths of 0.05			
	Odds Ratio (95% CI)	*p*-value	Odds Ratio (95% CI)	*p*-value		
Venous thromboembolism	0.921 (0.881–0.964)	0.0004*	0.925 (0.884–0.968)	0.0008*		
Myocardial infarction	1.984 (1.451–2.714)	< 0.0001*	2.054 (1.496–2.819)	< 0.0001*		
Urinary tract infection	1.091 (1.031–1.154)	0.002	1.095 (1.035–1.158)	0.0016		
Cognitive dysfunction	1.232 (1.135–1.337)	< 0.0001*	1.216 (1.121–1.320)	< 0.0001*		
Pneumonia	1.055 (0.998–1.115)	0.059	1.057 (0.999–1.112)	0.052		
In-hospital mortality	1.303 (1.209–1.404)	< 0.0001*	1.288 (1.196–1.387)	< 0.0001*		

*CI* means confidence interval

^*^p-values of < 0.001 are considered significant by the χ^2^ test

**Fig. 2:**
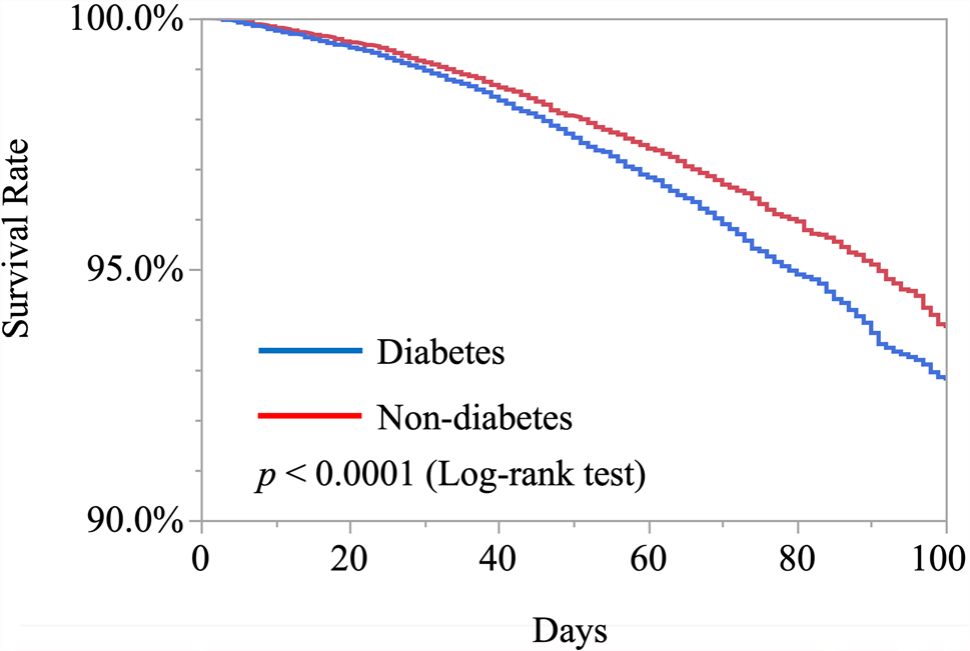
Survival rates of type 2 diabetes and non-diabetes groups. Results are expressed in the Kaplan–Meier curve. The 90-day survival rates were 93.7% in the type 2 diabetes group and 95.1% in the non-diabetes group. p < 0.0001 by log-rank test

Figure 2 shows the survival rates after surgery for hip fractures. The 90-day survival rate was 95.1% in the non-diabetes patient group and 93.7% in the type 2 diabetes patient group. The cumulative mortality rate differed significantly between the groups and the Kaplan–Meier survival curves also showed a significant difference between the type 2 diabetes group and the non-diabetes group throughout the follow-up period (log-rank test, p < 0.0001).

## Discussion

This nationwide retrospective cohort study investigated the impact of type 2 diabetes on postoperative complications and in-hospital mortality in elderly patients undergoing surgery for hip fractures in Japan. This study revealed that 18.5% of elderly Japanese patients with hip fractures had comorbid type 2 diabetes. After adjusting for potential confounders through propensity score matching, type 2 diabetes was significantly associated with increased risks of postoperative myocardial infarction, cognitive dysfunction, and in-hospital mortality. However, the absolute RDs for these outcomes were small, ranging from 0.0007 to 0.0045, indicating that while type 2 diabetes is statistically associated with adverse outcomes, its independent contribution to postoperative risk may be modest in clinical terms. These findings suggest that type 2 diabetes is a measurable, though relatively limited, risk factor for adverse postoperative outcomes in this patient population.

Our results align with previous studies that reported elevated postoperative mortality and complication rates among hip fracture patients with type 2 diabetes [16, 17]. In the present study, type 2 diabetes was significantly associated with in-hospital mortality (OR: 1.303, 95% CI: 1.209–1.404), postoperative myocardial infarction (OR: 1.984, 95% CI: 1.451–2.714), and cognitive dysfunction (OR: 1.232, 95% CI: 1.135–1.337). However, the absolute risk differences for these outcomes were small—0.0045, 0.0007, and 0.0029, respectively—and the number needed to harm (NNH) for in-hospital mortality was 200. These findings suggest that while type 2 diabetes is statistically associated with worse outcomes, the clinical impact per individual remains modest.

Despite the modest magnitude of risk increase, the high prevalence of type 2 diabetes in the elderly population may lead to a meaningful cumulative burden at the population level. The limited individual-level impact observed in our study may reflect improvements in perioperative glycemic control, standardized postoperative care protocols, and advances in surgical and anesthetic techniques [28–30]. In addition, the widespread implementation of early surgical intervention for hip fractures and the routine use of pharmacologic thromboprophylaxis have likely contributed to reducing the risk of venous thromboembolism and other complications in both diabetic and non-diabetic patients [18–20]. Furthermore, in the present analysis, major comorbidities associated with diabetes—including ischemic heart disease, cerebrovascular disease, renal impairment, and cognitive decline—were adjusted for through propensity score matching, allowing for a more accurate estimation of the independent effect of type 2 diabetes. These factors may mitigate the adverse effects of diabetes in acute care settings. Therefore, individualized perioperative strategies and risk stratification remain essential to optimizing outcomes in this vulnerable patient group.

Type 2 diabetes is associated with an increased risk of fractures, even in individuals with normal or high bone mineral density [31–33]. Studies have shown that among older adults and postmenopausal women, patients with type 2 diabetes typically have a 20–50% higher estimated fracture risk compared to control populations [31, 34, 35]. For example, one large cohort study reported a 24% increased risk of hip fractures among insulin users, while other studies have found relative risks ranging from 1.20 to 1.49 [35, 36]. Although several investigations have documented that patients with type 2 diabetes tend to have similar or even higher bone mineral density, numerous reports have paradoxically indicated an increased frequency of fractures, likely due to impaired bone quality rather than bone density alone [33, 34, 37].

From a clinical standpoint, our findings highlight the potential value of enhanced perioperative management in elderly hip fracture patients with type 2 diabetes. Although the absolute risk increase was modest, targeted strategies such as preoperative optimization of glycemic control, vigilant monitoring for signs of infection, and appropriate cardiac evaluation may be beneficial, particularly for patients with poorly controlled diabetes or multiple comorbidities. Additionally, efforts to minimize hospital stay and prevent immobilization-related complications could help mitigate postoperative risks in this vulnerable population.

One of the key methodological strengths of this study lies in the use of propensity score matching, which enabled us to reduce selection bias and control for confounding variables when comparing patients with and without type 2 diabetes. By matching a wide range of clinically relevant covariates, we aimed to approximate the conditions of a randomized controlled trial within the context of a large observational dataset. In addition, our focus on elderly Japanese patients addresses an important population-specific gap in the current literature, as ethnic and healthcare system differences may influence postoperative outcomes. These methodological features enhance the internal validity and clinical relevance of our findings.

There are several limitations to this large cohort study, which will be discussed below. First, the study population included patients with hip fractures who were treated exclusively in acute care hospitals and reported in the DPC data system. This excludes patients admitted to non-DPC-reported beds, which represent 30% of all general hospital beds, or patients never treated in an acute hospital [38]. Secondly, the limitations of this study include the inability to validate the names of DPC diseases and the inability to assess the severity of symptoms in actual patients. Third, the DPC database lacks granular data on glycemic control (e.g., HbA1c), diabetes duration, and specific antidiabetic treatments, which may influence postoperative outcomes. The improvement of diabetes-related bone fragility through advances in pharmacological treatment for type 2 diabetes represents an important and promising area for future research. Fourth, although conservative treatment may be chosen instead of surgery for elderly patients with hip fractures, this study only includes patients with surgical treatment, so the outcomes of conservative treatment in elderly patients with type 2 diabetes are unexplored. Finally, another limitation of the study is that the risk of death in the long term after discharge from the hospital was not assessed; further large-scale studies based on real patient data are needed.

### Conclusion

In conclusion, while type 2 diabetes was statistically associated with increased postoperative complications and in-hospital mortality in elderly patients undergoing hip fracture surgery, the absolute risk differences were small. These findings underscore the need for careful risk stratification and the selective application of tailored perioperative care strategies, particularly for patients with poorly controlled diabetes or multiple comorbidities. Future studies incorporating clinical and biochemical markers of diabetes control may help refine individual risk prediction and optimize intervention strategies.

## Supplementary Information

Below is the link to the electronic supplementary material.

Supplementary file1 (DOCX 32 KB)

## Data Availability

The datasets used and/or analyzed during the current study are available from the corresponding author upon reasonable request.

## Abbreviations

BMI: Body mass index
CI: Confidence interval
DPC: Diagnosis procedure combination
OR: Odds ratio
RD: Risk difference
SERM: Selective estrogen receptor modulator
SMD: Standard mean difference
