# Supplementary material for: Postoperative risks of type 2 diabetes in elderly hip fracture patients: a propensity score-matched study
Source: J Bone Miner Metab. 2025 Jul 1;43(5):553–63. doi: 10.1007/s00774-025-01624-9 (PMC12620317; doi:10.1007/s00774-025-01624-9)
Supplement: Supplementary file 1 — Supplementary file1 (DOCX 32 KB) [file 774_2025_1624_MOESM1_ESM.docx]

Supplemental Table 1 Comparison of postoperative complications, length of hospital stay, and transfusion volume between patients with and without type 2 diabetes in subgroup analyses of femoral neck and trochanteric fractures

|  | Femoral neck fracture | | | Trochanteric fracture | | |
| --- | --- | --- | --- | --- | --- | --- |
|  | Non-diabetes  (n = 43551) | Diabetes  (n = 42757) | *p*-value | Non-diabetes  (n = 37735) | Diabetes  (n = 38624) | *p*-value |
| Venous thromboembolism | 2045 (4.7%) | 1894 (4.4%) | 0.061 | 1970 (5.2%) | 1829 (4.7%) | 0.002 |
| Myocardial infarction | 23 (0.05%) | 59 (0.14%) | < 0.0001* | 36 (0.1%) | 57 (0.15%) | 0.039 |
| Urinary tract infection | 1162 (2.7%) | 1297 (3.0%) | 0.0013 | 1173 (3.1%) | 1245 (3.2%) | 0.36 |
| Cognitive dysfunction | 500 (1.2%) | 637 (1.5%) | < 0.0001* | 526 (1.4%) | 630 (1.6%) | 0.007 |
| Pneumonia | 1194 (2.7%) | 1269 (3.0%) | 0.046 | 1254 (3.3%) | 1299 (3.4%) | 0.76 |
| In-hospital mortality | 540 (1.2%) | 722 (1.7%) | < 0.0001* | 679 (1.8%) | 838 (2.2%) | 0.0002* |
| Length of hospitalization (days) | 35.2 ± 28.2 | 36.7 ± 28.5 | < 0.0001* | 36.5 ± 27.5 | 37.4 ± 31.9 | < 0.0001* |
| Blood transfusion Day 0 (unit) | 0.25 ± 0.80 | 0.26 ± 0.82 | 0.34 | 0.55 ± 1.17 | 0.52 ± 1.14 | 0.0001* |
| Blood transfusion Day 1 (unit) | 0.15 ± 0.59 | 0.16 ± 0.61 | 0.006 | 0.41 ± 0.94 | 0.39 ± 0.92 | 0.0006* |
| **p*-values of < 0.001 are considered significant by the χ^2^ test and Student’s t-test. | | | | | | |
